# Oxaloacetate induces apoptosis in HepG2 cells via inhibition of glycolysis

**DOI:** 10.1002/cam4.1410

**Published:** 2018-03-13

**Authors:** Ye Kuang, Xiaoyun Han, Mu Xu, Qing Yang

**Affiliations:** ^1^ Department of Pathogenobiology College of Basic Medical Sciences Jilin University 126 Xinmin Street Changchun 130021 Jilin Province China

**Keywords:** Akt/HIF pathway, apoptosis, glycolysis, oxaloacetate, oxidative phosphorylation

## Abstract

Most cancer cells perform glycolysis despite having sufficient oxygen. The specific metabolic pathways of cancer cells have become the focus of cancer treatment. Recently, accumulating evidence indicates oxidative phosphorylation (OXPHOS) and glycolysis can be regulated with each other. Thus, we suggest that the glycolysis of cancer cells is inhibited by restoring or improving OXPHOS in cancer cells. In our study, we found that oxaloacetate (OA) induced apoptosis in HepG2 cells in vivo and in vitro. Meanwhile, we found that OA induced a decrease in the energy metabolism of HepG2 cells. Further results showed that the expression and activity of glycolytic enzymes were decreased with OA treatment. Conversely, the expression and activity of enzymes involved in the TCA cycle and OXPHOS were increased with OA treatment. The results indicate that OA can inhibit glycolysis through enhancement of OXPHOS. In addition, OA‐mediated suppression of HIF1α, p‐Akt, and c‐myc led to a decrease in glycolysis level. Therefore, OA has the potential to be a novel anticancer drug.

## Introduction

The abnormal metabolism of cancer cells has become the focus of cancer therapy 1, 2, 3, 4. Compared to normal cells, cancer cells rely too much on glycolytic pathway for ATP generation despite having sufficient available oxygen to support oxidative phosphorylation (OXPHOS). This phenomenon is known as the Warburg effect 5, 6, 7, 8, 9. In fact, as a heterogeneous disease, each cancer type has unique metabolic characteristics 10. Contrary to the Warburg effect, some cancer cells are known to obtain ATP mainly through OXPHOS 11, 12. For example, OXPHOS is very active in human cervical carcinoma (HeLa) cells, human leukemia cell (HL60) cells, and human osteosarcoma cell (143B) cells 13. Thus, we hypothesize that OXPHOS and glycolysis must be coordinated to maintain energy balance in cancer cells. To maintain the energy balance in cancer cells, glycolysis must be enhanced if OXPHOS is weakened, and in turn, glycolysis must be weakened if OXPHOS is enhanced. Indeed, recently published papers report that metabolic switch between OXPHOS and glycolysis can be regulated 14, 15, 16, 17, 18.

The tricarboxylic acid (TCA) cycle is a metabolic center of OXPHOS and fulfills the biosynthetic, bioenergetic, and redox balance requirements of cells 19, 20. Oxaloacetate (OA) is an important intermediate in the TCA cycle and participates in the metabolism of energy production. Pyruvate is oxidized to acetyl‐CoA and then condensed into citric acid with OA in the presence of oxygen 21, 22. This is the beginning of TCA cycle. Recent research has shown that adding OA to cultured neuronal SHSY5Y cells can enhance respiratory fluxes 23. Other research suggests that OA is able to activate brain mitochondrial biogenesis 24. We suggest that OA can enhance the OXPHOS level in the cells. Thus, we further pose a hypothesis that OA is able to inhibit aerobic glycolysis by increasing OXPHOS in cancer cells.

Although changes in metabolites can regulate cellular metabolism, genes, and related signaling pathways also affect metabolic processes in cancer cells 25, 26. The Akt/HIF signaling pathway regulates aerobic glycolysis through transcriptional activation of HIF1α, NFκB, c‐myc, and the subsequent expression of glycolytic enzymes such as hexokinase 2 (HK2), phosphofructokinase 2 (PFK2), and pyruvate kinase isozyme type M2 (PKM2) 27, 28, 29, 30, 31. In our research, we found OA inhibited the expression of glycolytic enzymes through inhibition of the Akt/HIF signaling pathway.

In our research, we confirmed the anticancer effect of OA in vivo and in vitro. We found that the OA‐mediated apoptosis in cancer cells was related to the inhibition of aerobic glycolysis. We further found that OA inhibited glycolysis via enhancement of OXPHOS and suppression of the Akt/HIF pathway. In addition, our preliminary study revealed that OA selectively inhibited cancer cells with high levels of glycolysis.

## Materials and Methods

### Cell lines and reagents

Human hepatocellular liver carcinoma cell line (HepG2) and human normal liver cell line (LO‐2) were obtained directly from the cell bank of Shanghai Institute of Life Sciences (China). HepG2 cells and LO‐2 cells were cultured in DMEM medium supplemented with 10% fetal bovine serum (FBS) and 1% penicillin‐streptomycin (100 IU/mL penicillin and 100 mg/mL streptomycin) in a 5% carbon dioxide (CO_2_) humidified air atmosphere at 37°C. Cell culture reagents (FBS and DMEM) were purchased from HyClone (Logan, Utah, USA, 10091130, 1249101). Oxaloacetic acid was purchased from Sigma (St. Louis, MO, 328‐42‐7). Oxaloacetic acid powder was dissolved in phosphate‐buffered saline (PBS) and pH adjusting to approximately 7.0 with NaOH. Because OA was relatively unstable in solution and the pH gradually increased over 2 h, it must be with the current use.

### Tumor xenograft model

Four‐week‐old female BALB/cA nude mice (*n* = 30) were purchased from the Experimental animal center of the Chinese Academy of Sciences Shanghai (China) and raised in the SPF‐class laboratory. The study protocol was approved by the Ethics committee of the Jilin University Health Science Center. During the experiment, animal handling and care were carried out according to the National Institutes of Health Guide for the Care and Use of Laboratory Animals (NIH Publications No. 8023, revised 1978). The mice were randomly divided into two groups (15 in a group). The method of back inoculation was used in nude mice. When the tumor volume reached approximately 500 mm^3^, PBS (10 μL/g) (control group) or OA solution (50 mmol/L, 10 μL/g) (OA group) was injected into the xenograft tumors once every 3 days. Tumor size and mice weight were respectively measured by a digital caliper and electronic balance every 2 days. The tumor volume was calculated using the following formula: *V* = (length × width^2^)/2. The tumors were removed, photographed, and weighed after all the mice were sacrificed.

### Patients and tissue specimens

In our research, we collected 20 cases of tumor samples from liver cancer patients in the Third Hospital of Jilin University (China) between October 2015 and June 2016. This study was approved by the Ethics Committee of School of Basic Medical Sciences, Jilin University. All patients were consented and signed an informed consent form.

### Primary hepatoma carcinoma cells

The patients’ liver cancer tissues were rinsed with Hanks liquid. Then, the tissues were cut into 1~2 cm^3^ small pieces by ophthalmic scissors. The tissues were digested 30 min at 37°C. The digestive juices were got rid of the tissue blocks through 100 mesh aperture of stainless steel wire mesh filter. The filtrate was removal of trypsin in 150–200 g for 3~5 min and then was rinsed with Hanks liquid twice. After counting cells and trypan blue staining, the cells were cultured in DMEM supplemented with 1% penicillin‐streptomycin and 10% FBS in a 5% CO_2_ and 37°C condition.

### Mutant version of HIF1α

To prevent HIF1α from being degraded under normoxic conditions, a vector containing mutant version of HIF1α with double proline‐to‐alanine substitutions at 402 and 564 was used from Addgene (http://https://www.addgene.org/19005/). The coding gene of HIF1α with the double mutations in the mutant vector was cleaved by BamHI and HindIII. The gene segment was ligated into pcDNA3.0 to construct a pcDNA3.0‐mut‐HIF1α expression vector. The pcDNA3.0‐mut‐HIF1α expression vector was transiently transfected into HepG2 cells using Lipofectamine 2000 (Invitrogen, Carlsbad, CA, 11668019). The empty expression vector pcDNA3.0 was transfected into HepG2 cells and used as a control.

### Cell viability and colony formation assay

Cell viability was measured using an MTT assay (Sigma, M2128). Briefly, cells were inoculated in 96‐well plates at a density of 8 × 10^3^ cells/mL. After 24 h, the cells were adherent with free serum for 24 h. To each well, the medium was replaced with serum‐free medium containing 10 μL MTT solution (5 mg/mL) and incubated at 37°C for 4 h. Formazan crystals were fully dissolved in DMSO, and the absorbance at 490 nm was detected by spectrophotometer (Thermo Scientific, Waltham, Massachusetts, USA). HepG2 cells were inoculated in six‐well plates at a density of 400 cells/mL. When most colonies contained more than 50 cells, the clones were immobilized with methanol for 30 min and stained with 0.5% crystal violet.

### Analysis of cell apoptosis

The apoptosis in HepG2 cells and primary hepatoma carcinoma cells was detected by flow cytometry (FCM) using the Annexin V: PI kit (BD, Franklin lakes, New Jersey, USA, 556463, 560931). Briefly, the cells were collected and washed twice with cold PBS. The cells were incubated in 100 μL of binding buffer with 5 μL Annexin V and 10 μL PI in the dark at room temperature for 30 min. The samples were analyzed by FCM (Beckman Coulter, Miami, FL). The caspase 3 activity of HepG2 cells was detected using caspase 3 activity assay kit (Jiancheng, China). Briefly, the cells were collected and washed twice with cold PBS. The cells were resuspended and lysed on ice for 30 min. The supernatant was incubated with Ac‐DEVD‐pNA and reaction buffer at 37°C for 4 h. The absorbance at 405 nm was recorded with a spectrophotometer (Thermo Scientific).

### Activity of metabolic enzymes measurements

According to the manufacturer's instructions, we measured the activity of hexokinase (HK), phosphofructokinase (PFK), lactate dehydrogenase (LDH), citroyl synthetase (CS), pyruvate dehydrogenase (PDH), and isocitrate dehydrogenase (IDH) using the assay kits from Sigma (MAK091, MAK093, MAK066, MAK193, MAK183, and MAK062) by spectrophotometer (Eppendorf, Germany). We measured the activity of mitochondrial respiratory chain complex I/II (COX I/II) using the assay kits from Jiancheng (China, A089‐1, A089‐2) by spectrophotometer.

### Western blot analysis

The cells were lysed with RIPA (Sigma, R0278) on ice for 30 min, and then the lysate was centrifuged at 4°C 12,000 *g* for 30 min. The supernatant was retained, and the protein concentration was detected using BCA method (Sigma, BCA1). The equal amount of protein was separated in SDS‐PAGE gel and transferred onto a polyvinylidene difluoride (PVDF) membrane (Bio‐Rad, Hercules, CA, 1620177). The membranes were blocked in skim milk for 3 h at room temperature and then were incubated with the primary antibody at room temperature for 2 h. The membranes were washed with Tris‐buffered saline containing Tween‐20 (TBST) three times each for 10 min and incubated with the secondary antibody for 1 h. The membranes were washed with TBST three times each for 10 min again. Finally, the protein bands were exposed using enhanced chemiluminescence (ECL) (Proteintech, Wuhan, Hubei, China, B500024) by Image Quant LAS 4000 digital imaging system (GE, Fairfield, Connecticut, USA). The related antibodies against the following proteins were used: Bax (1:1000), Bcl‐2 (1:1000), COX I (NDUFB8) (1:1000), COX II (SDHB) (1:500), caspase 3 (1:500), PGC‐1α (1:1000), SIRT1 (1:800) (Abcam, Cambridge Science Park, Cambridge, UK, ab32503, ab32124, ab110242, ab14714, ab13847, ab54481, and ab110304), HIF1α (1:1000) (Genetex, GTX127309), Akt (1:500), p‐Akt (1:500), c‐myc (1:500), cleaved caspase 3 (1:1000), and cleaved PARP (1:1000) (Wanleibio, China, wl0003b, wlp001, wl0116, wl01857, and wl01932).

### TUNEL assay

TUNEL assay was used to detect apoptosis of xenograft tumor tissues. The detection kit was purchased from Beyotime (China). Briefly, paraffin section was prepared, dewaxed with dimethylbenzene, dehydrated with ethanol, and treated with DNase‐free protease K for at 37°C for 15–30 min. After washed twice with PBS, the paraffin section was incubated with 50 μL TUNEL detection solution at 37°C in dark for 1 h and then visualized with a fluorescence microscope (Olympus, B × 53, Japan). The percentage of apoptotic cells in tumor tissues was quantitatively calculated as the ratio of TUNEL‐positive cells (green) to total cell nuclei (blue). At least 300 cells were counted from five random fields by two observers from three independent experiments.

### RNA isolation and qRT‐PCR

Total RNA was extracted from cells using RNAiso Plus (TaKaRa, Japan, 108‐95‐2) and isolated according to the manufacturer's instructions. The RNA concentration and purity were measured by a BioSpectrometer (Eppendorf, Germany); 2 μg total RNA was reversely transcribed into cDNA using the TransScript RT reagent Kit (TransGen, China, AE301). According to the manufacturer's instructions, qRT‐PCR was performed with FastStart Universal SYBR Green Master (Vazyme, China, Q111) using a Gene Amp 9600 PCR system (Perkin‐Elmer, Waltham, MA). The relative amount of cDNA was analyzed using the 2^−ΔΔCT^ method. The primers for qRT‐PCR used in this study were as follows: PDHA1‐Forward: CTTACCGCTACCATGGACACAGCATG,

Reverse: CTCCTTTAATTCTTCAACACTTGCAAGA; HK2‐Forward: GAGCCACCACTCACCCTACT, Reverse: CCAGGCATTCGGCAATGTG; PFK2‐Forward: ATTGCGGTTTTCGATGCCAC, Reverse: GCCACAACTGTAGGGTCGT; IDH1‐ Forward: TTGGCTGCTTGCATTAAAGGTT, Reverse: GTTTGGCCTGAGCTAGTTTGA; CS‐Forward: GAGCAGGCCAGAGTTAAGAC, Reverse: AAAATAAGCCCTCAGGTAGG; LDHA‐Forward: AAACGCGCCTTAATTTAGTCCA, Reverse:CAGCCGCTTCCAATAATACGG; PGC1α‐Forward: GTAAATCTGCGGGATGATGG, Reverse: AGCAGGGTCAAAATCGTCTG; SIRT1‐Forward: TGCCATCATGAAGCCAGAGA, Reverse: AACATCGCAGTCTCCAAGGA; and GAPDH‐Forward:CAAGAAGGTGGTGAAGCAGG, Reverse: CCACCCTGTTGCTGTAGCC.

### ATP glucose, lactic acid measurements

ATP production of HepG2 cells was detected using an ATP Bioluminescent Assay Kit (LDEBIO, Guangzhou, Guangdong, China, 1001) according to the manufacturer's instructions. Glucose consumption of HepG2 cells was detected using a Glucose measurement Assay Kit (Rongsheng, China, 361500) according to the manufacturer's instructions. Lactic acid production of HepG2 cells was detected using the Assay Kit (Jiancheng, China, A020) according to the manufacturer's instructions.

### Classification of cancer cell lines for glycolysis

To identify glycolysis levels of different tumor cell lines, we performed unsupervised hierarchical clustering analysis on normalized log^2^‐transformed microarray data for 21 genes that made up the glycolysis metagene signature (TPI1, PGM2, PGM1, PGAM2, PFKP, PDHA2, PCK2, LDHA, HK2, HK1, G6PC, FBP2, FBP1, ENO, ALDOC, ALDOB, ALDH3B2, ALDH3A2, ALDH3A1, ALDH2, and ADH6). Microarray gene expression data of those cancer cell lines were downloaded from one GEO dataset. The series number is GSE57083. Unsupervised hierarchical clustering analysis was applied with Euclidean distance and complete linkage.

### Statistical analysis

All experimental data were presented as the mean ± standard deviation (SD) of at least three independent experiments (SPSS, IBM, Armonk, New York, USA). Data comparing between two groups were statistically analyzed by two‐tailed *t*‐test. Only results with *P* < 0.05 were considered to be statistically significant: *P < 0.05, **P < 0.01, ***P < 0.001.

## Results

### OA induces apoptosis in HepG2 cells

**Figure 1:**
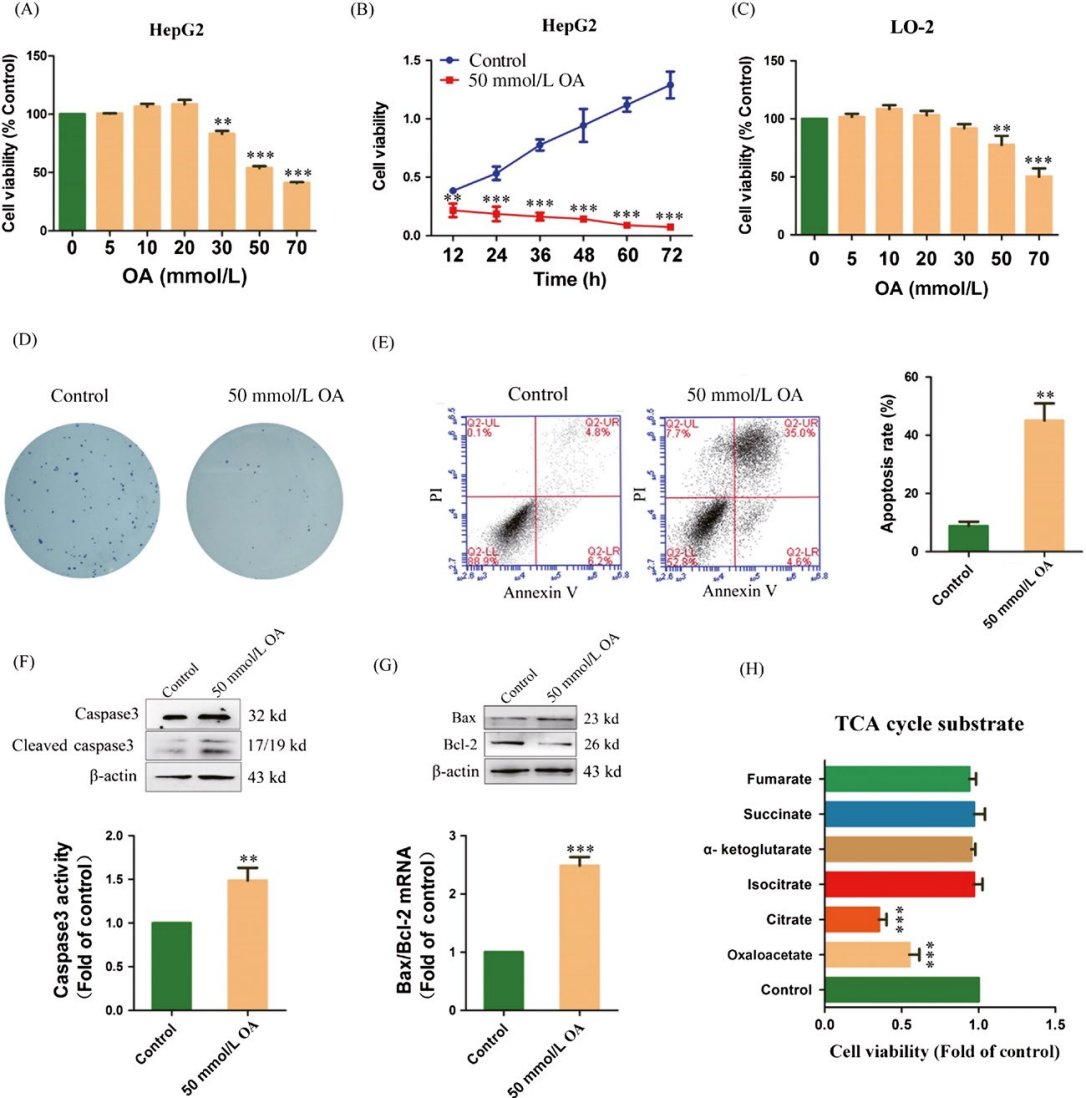
Oxaloacetate (OA) induced apoptosis in HepG2 cells. (A) HepG2 cells were treated with different concentrations of OA for 24 h. The cell viability was measured using an MTT assay. (B) The MTT assay was performed at 12, 24, 36, 48, 60, and 72 h in HepG2 cells treated with 50 mmol/L OA. (C) LO‐2 cells were treated with different concentrations of OA for 24 h. The cell viability was measured using an MTT assay. (D) Clone formation assay. (E) Apoptosis in HepG2 cells was detected by FCM using the Annexin V: PI assay. Histograms depict the proportions of total apoptotic cells (F) caspase 3 protease activity was measured using a caspase 3 assay kit. The protein expression of caspase 3 was measured using Western blot. (G) The mRNA expression and protein expression of Bax and Bcl‐2 were detected using qRT‐PCR and Western blot. (H) HepG2 cells were treated with different TCA cycle substrates (50 mmol/L) for 24 h. The cell viability was measured using an MTT assay. **P < 0.01, ***P < 0.001 versus control. The results expressed as the mean ± SD of three independent experiments.

To determine whether OA impacted cancer cell survival, we treated HepG2 cells with a gradient dosage of OA (5–70 mmol/L) for 24 h. HepG2 cells in the control group were treated with equal dose of PBS. The data showed that treatment with 50 mmol/L or 70 mmol/L OA resulted in a significant decrease in the viability of HepG2 cells (Fig. 1A). The IC50 value of OA against HepG2 cells after being treated for 24 h was 56.2 ± 1.03 mmol/L. As shown in Figure 1B, we found a time‐dependent decrease in viability of cells treated with 50 mmol/L OA. We further detected whether OA had toxicity to normal cells. LO‐2 cells were treated with a gradient dosage of OA (5–70 mmol/L) for 24 h. The data showed that 50 mmol/L OA had slight toxicity to LO‐2 cells (77.56 ± 7.89% of the control) (Fig. 1C). Therefore, 50 mmol/L OA was used in the subsequent experiments. Colony formation assays indicated that the colony number in HepG2 cells treated with 50 mmol/L OA was much lower than that of the control (Fig. 1D). We also carried out an analysis of apoptosis and found that the rate of apoptosis was increased in HepG2 cells treated with 50 mmol/L OA for 24 h compared to the control (5.25 ± 1.16‐fold of the control) (Fig. 1E). We further examined the expression of apoptotic genes in HepG2 cells. Compared to the control, HepG2 cells highly expressed cleaved caspase 3 when treated with 50 mmol/L OA for 24 h (Fig. 1F). The activity of caspase 3 was also increased to 1.42 ± 0.13‐fold of the control in HepG2 cells (Fig. 1F). The ratio of Bax and Bcl‐2 mRNA expression in HepG2 cells was increased to 2.48 ± 0.16‐fold of the control when treated with 50 mmol/L OA (Fig. 1G). Finally, we examined whether the inhibitory effect of OA in HepG2 cells was unique to the TCA cycle substrate. The results showed that only OA and citrate had a significant inhibitory effect on the TCA cycle substrate (55.12 ± 6.55 and 35.33 ± 4.93% of the control) (Fig. 1H). In summary, these data indicated that OA could induce apoptosis in HepG2 cells.

### Effects of OA on glycolysis

**Figure 2:**
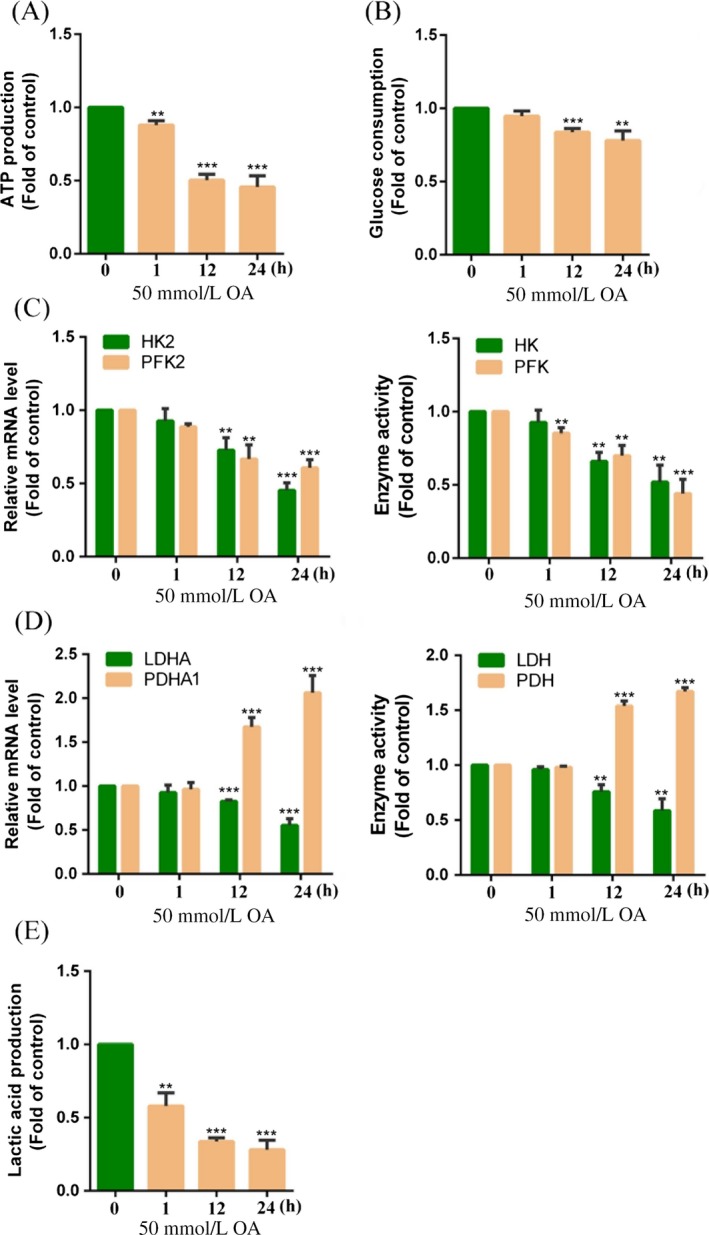
Oxaloacetate (OA) inhibited glycolysis in HepG2 cells. HepG2 cells were treated with 50 mmol/L OA for 1, 12, and 24 h. (A) ATP production was detected using an ATP assay kit. (B) Glucose consumption was detected using a glucose assay kit. (C) The mRNA expression of HK2 and PFK2 as well as activity of HK and PFK was detected using qRT‐PCR and the assay kits. (D) The mRNA expression of LDHA and PDHA1 as well as activity of LDH and PDH was detected using qRT‐PCR and the assay kits. (E) Lactic acid production was detected using a lactic acid assay kit. **P < 0.01, ***P < 0.001 versus control. The results expressed as the mean ± SD of three independent experiments.

Considering that OA is a TCA intermediate, we suggest that the anticancer effect of OA is related to cellular energy metabolism. Thus, ATP production and glucose consumption were detected in HepG2 cells. The results showed that ATP production was decreased compared to the control (0 h) in HepG2 cells treated with 50 mmol/L OA for 1, 12, and 24 h (88.35 ± 3.21, 50.31 ± 4.01, and 45.61 ± 7.61% of the control) (Fig. 2A). The glucose consumption was also decreased compared to the control (0 h) in HepG2 cells treated with 50 mmol/L OA for 1, 12, and 24 h (94.66 ± 3.51, 83.62 ± 2.35, and 78.22 ± 6.45% of the control) (Fig. 2B). We suggest that the decrease in energy metabolism is due to the inhibition of aerobic glycolysis. The mRNA expression and activity of key enzymes in glycolysis were further detected in HepG2 cells. The results showed that the mRNA expression of HK2 and PFK2 as well as the activity of HK and PFK was obviously decreased compared to the control (0 h) in HepG2 cells treated with 50 mmol/L OA for 12 and 24 h (45.36 ± 5.16 and 60.61 ± 5.57% of the control; 52.91 ± 11.57 and 44.86 ± 9.83% of the control at 24 h) (Fig. 2C). These results also showed that OA could induce a decrease in glycolysis in HepG2 cells. We next detected the mRNA expression of LDHA and PDHA1 as well as the activity of LDH and PDH in HepG2 cells. As shown in Figure 2D, the mRNA expression of LDHA and the activity of LDH were obviously decreased compared to the control (0 h) in HepG2 cells with treated 50 mmol/L OA for 12 and 24 h (55.32 ± 7.51 and 58.65 ± 1.23% of the control at 24 h). In contrast, the mRNA expression of PDHA1 and the activity of PDH were increased compared to the control (2.03 ± 1.1 and 1.61 ± 0.03‐fold of the control at 24 h). Compared to the control (0 h), lactic acid production was also obviously decreased in HepG2 cells treated with 50 mmol/L OA for 1, 12, and 24 h (58.16 ± 8.82, 33.6 ± 2.57, and 28.1 ± 6.53% of the control) (Fig. 2E). We suggest that OA can inhibit glycolysis and induce a shift from glycolysis to OXPHOS in HepG2 cells.

### Effects of OA on the TCA cycle and OXPHOS

**Figure 3:**
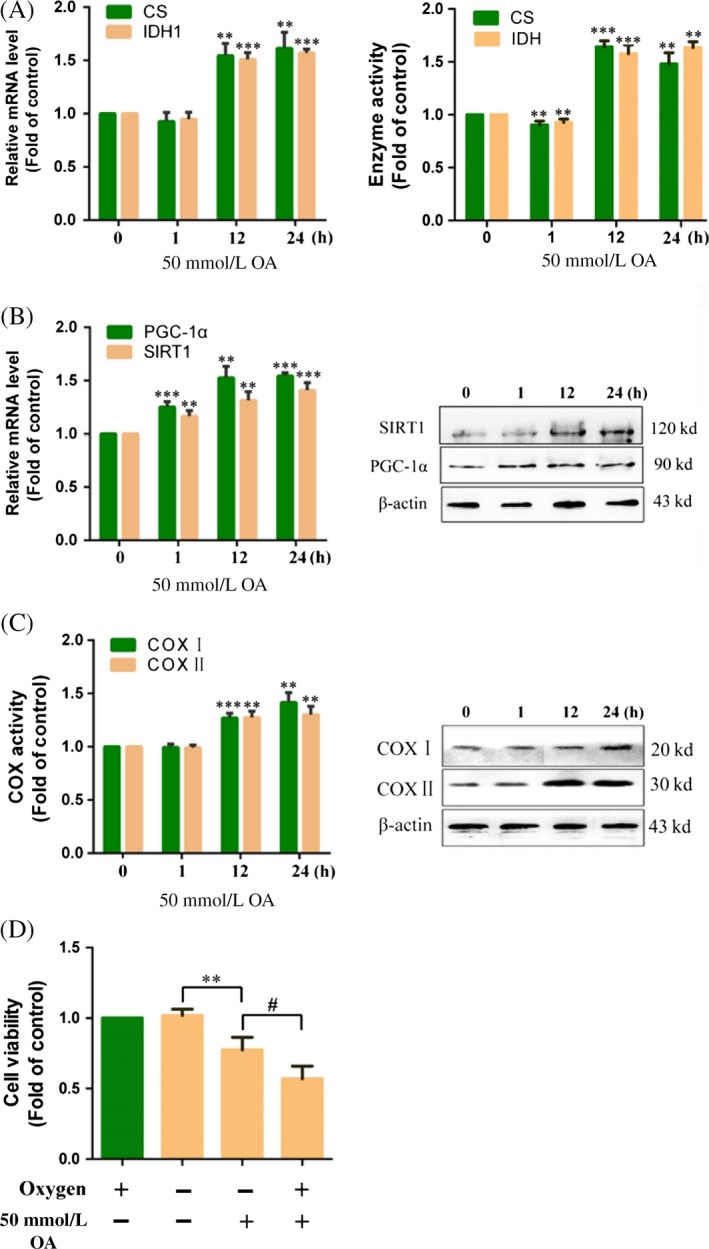
Oxaloacetate (OA) promoted TCA cycle and OXPHOS. HepG2 cells were treated with 50 mmol/L OA for 1, 12, and 24 h. (A) The mRNA expression of CS and IDH1 as well as activity of CS and IDH was detected using qRT‐PCR and the assay kits. (B) The mRNA expression and protein expression of PGC‐1α and SIRT1 were detected using qRT‐PCR and Western blot. (C) The activity and protein expression of COX I and COX II were detected using the assay kits and Western blot. (D) HepG2 cells were treated 50 mmol/L OA for 24 h in hypoxia or normoxia. The cell viability was measured using an MTT assay. **P < 0.01, ***P < 0.001 versus control. ^#^ *P* < 0.05 versus normoxia group. The results expressed as the mean ± SD of three independent experiments.

Previous studies have shown that OA causes a decrease in glycolysis levels. We hypothesize that OA induces inhibition of glycolysis through enhancement of OXPHOS in HepG2 cells. Therefore, we detected the mRNA expression and activity of key enzymes in the TCA cycle in HepG2 cells. The results showed that the mRNA expression of CS and IDH1 as well as the activity of CS and IDH was obviously increased compared to the control (0 h) in HepG2 cells treated with 50 mmol/L OA for 12 and 24 h (1.61 ± 0.15 and 1.57 ± 0.03‐fold of the control; 1.48 ± 0.18 and 1.73 ± 0.09‐fold of the control at 24 h) (Fig. 3A). Here, we confirm that OA is able to drive the TCA cycle. PGC‐1α is a master regulator of mitochondrial biogenesis and energy metabolism. SIRT1 can activate PGC‐1α by deacetylation. We further detected the role of PGC‐1α and SIRT1 on OA‐induced activation of OXPHOS. As shown in Figure 3B, compared to the control (0 h), the mRNA expression and protein expression of PGC‐1α and SIRT1 were increased in HepG2 cells treated with 50 mmol/L OA. We further detected the protein expression and activity of COX I and COX II in HepG2 cells. The results showed that the protein expression and activity of COX I and COX II were increased compared to the control (0 h) in HepG2 cells treated with 50 mmol/L OA for 12 and 24 h (1.41 ± 0.09 and 1.31 ± 0.07‐fold of the control at 24 h) (Fig. 3C). To further determine whether OA inhibited glycolysis by promoting OXPHOS**,** we observed the anticancer effect of OA under hypoxic condition. As shown in Figure 3D, when HepG2 cells were treated with 50 mmol/L OA for 24 h, the cell viability under normoxia conditions was 75.19 ± 19.52% that of hypoxia. The results indicate the anticancer effect of OA is related to OXPHOS in HepG2 cells.

### Effects of OA on tumor growth in vivo

**Figure 4:**
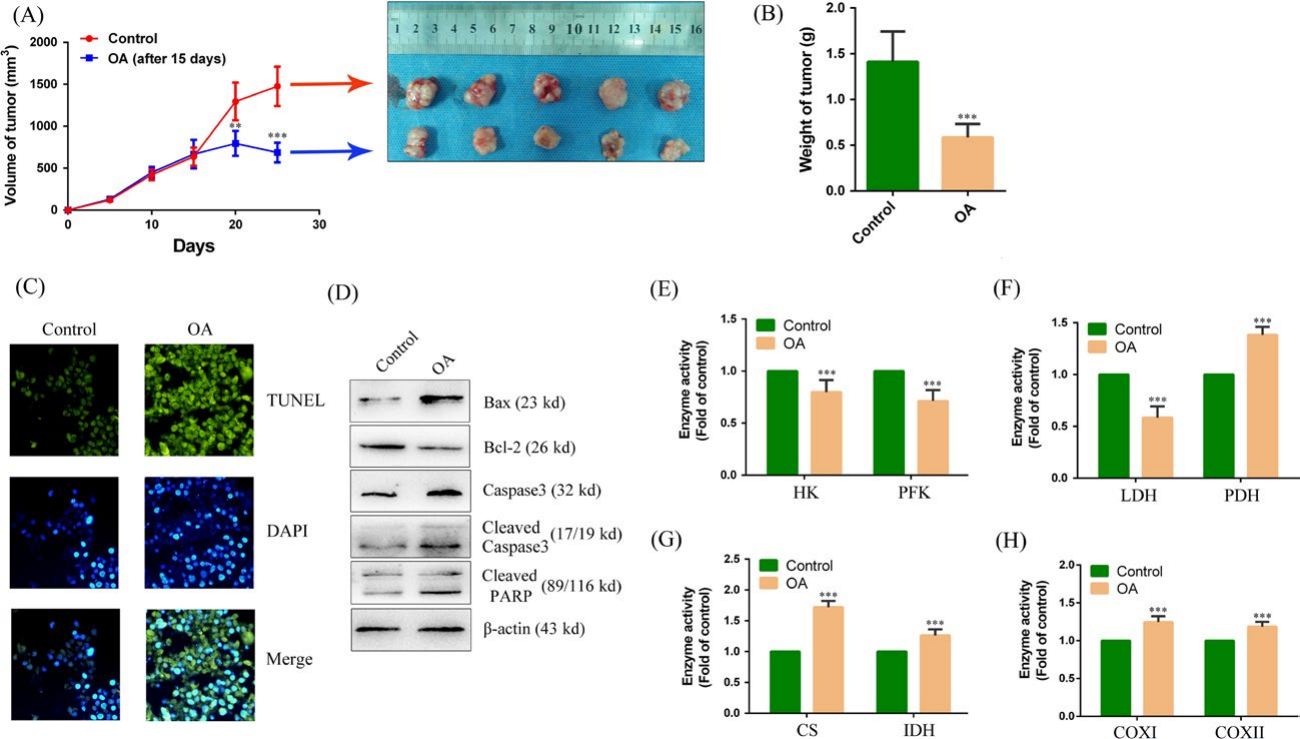
Oxaloacetate (OA) inhibited tumor growth in vivo. (A) Time‐dependent tumor volumes in the control and the OA group. Photographs of tumors derived from nude mice in the control and the OA group. (B) Average weight of tumors from nude mice. (C) TUNEL assay to detect the apoptosis in tissue. TUNEL‐positive cells (green). DAPI was localization nucleus (Blue). The merge image was overlay of TUNEL and DAPI image. All the image magnification was 200 × . (D) Western blot assay. The protein expression of Bax, Bcl‐2, caspase 3, cleaved caspase 3, and cleaved PARP in tissue. (E–H) The activity of HK, PFK, LDH, PDH, CS, IDH, COX I, and COX II was detected on tumors using the assay kits. **P < 0.01, ***P < 0.001 versus control. The results expressed as the mean ± SD of three independent experiments.

We used a xenograft model to identify whether the in vitro anticancer effect of OA would also be true in vivo. As shown in Figure 4A, tumors in the OA‐treated group grew more slowly than those in the control group. Additionally, portions of OA‐treated tumors appeared necrotic. Tumors excised from OA‐treated animals were smaller than tumors from the control group (Fig. 4A). The average weight of the tumors derived from the OA group was also lower than that of the tumors derived from the control group (42.76 ± 11.99% of the control) (Fig. 4B). The results of TUNEL assay showed that the tumor tissues in the OA‐treated group had a significant increase in apoptosis than did the control group. Percentage of the apoptotic cells was 72.38 ± 8.43% (mean ± SD) in the OA‐treated group that had a 4.33 ± 3.37‐fold greater increase in the percentage than did the control group (*P *<* *0.001) (Fig. 4C). In the Western blot experiments, the tumor tissues in the OA‐treated group had a significant increase in the protein level of Bax/Bcl‐2, cleaved caspase 3 and cleaved PARP than did the control group (Fig. 4D), indicating increased apoptosis in tissues in the OA‐treated group. We further detected the activity of enzymes related to energy metabolism in the tumors. The results showed that the activity of HK, PFK, and LDH in the OA group was decreased when compared to the control group (79.85 ± 11.49, 71.28 ± 10.39, and 58.42 ± 10.76% of the control) (Fig. 4E and F). In contrast, the activity of CS, IDH, PDH, COX I, and COX II in the OA group was increased when compared to the control group (1.72 ± 0.12, 1.26 ± 1.21, 1.38 ± 0.09, 1.25 ± 0.07, and 1.18 ± 0.06‐fold of the control) (Fig. 4F–H). These results further confirm that OA induces apoptosis in liver cancer in vivo.

### Effects of OA in primary hepatoma carcinoma cells

**Figure 5:**
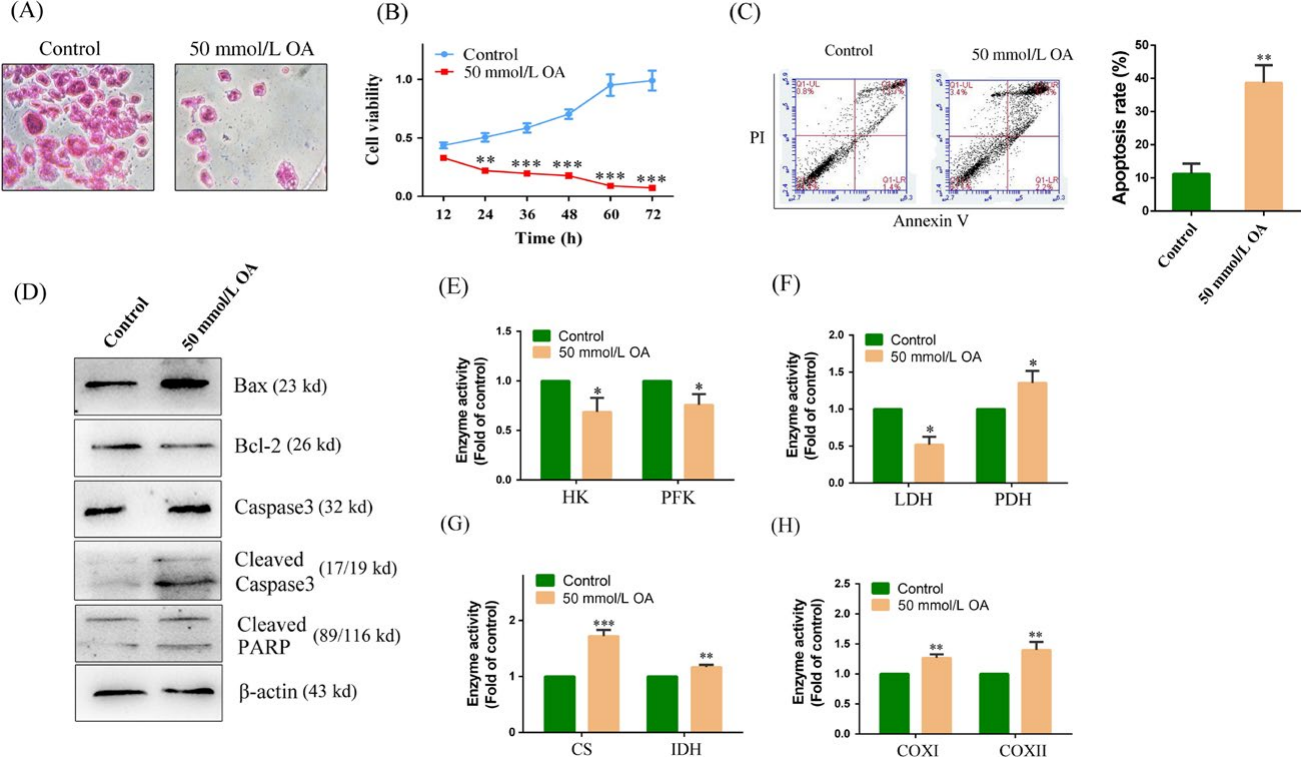
Oxaloacetate (OA) induced apoptosis in primary hepatoma carcinoma cells. Primary hepatoma carcinoma cells were treated with 50 mmol/L OA for 24 h. (A) HE staining. The image magnification was 200 × . (B) Cell growth curve was detected using an MTT assay. (C) Apoptosis in primary hepatoma carcinoma cells was detected by FCM using the Annexin V/PI assay. The histogram depicts the proportions of total apoptotic cells. (D) Western blot assay. The protein expression of Bax, Bcl‐2, caspase 3, cleaved caspase 3, and cleaved PARP in primary hepatoma carcinoma cells. (E)–(H) The activity of HK, PFK, LDH, PDH, CS, IDH, COX I, and COX II was detected in primary hepatoma carcinoma cells using the assay kits.*P < 0.05, **P < 0.01, ***P < 0.001 versus control. The results expressed as the mean ± SD of three independent experiments.

To further clarify the anticancer effect of OA, we conducted a primary culture of fresh liver cancer tissue and observed the anticancer effect of OA. As shown in Figure 5A, when primary hepatoma carcinoma cells were treated with 50 mmol/L OA for 24 h, the cells appeared apoptosis and the number of cells was decreased under the microscope. MTT assay results showed a time‐dependent decrease in cell viability when primary hepatoma carcinoma cells were treated with 50 mmol/L OA (Fig. 5B). The results of Annexin V/PI assay showed that the primary hepatoma carcinoma cells treated with 50 mmol/L OA had a significant increase in the apoptosis rate than did the control cells (3.64 ± 0.96‐fold of the control) (Fig. 5C). In the Western blot experiments, the primary hepatoma carcinoma cells treated with 50 mmol/L OA had a significant increase in the protein level of Bax/Bcl‐2, cleaved caspase 3 and cleaved PARP than did the control cells (Fig. 5D), indicating increased apoptosis in these OA‐treated cells. We further detected the activity of enzymes related to energy metabolism in primary hepatoma carcinoma cells. The results showed that the activity of HK, PFK, and LDH was decreased compared to the control group in primary hepatoma carcinoma cells treated with 50 mmol/L OA for 24 h (68.65 ± 14.29, 75.33 ± 11.59, and 52.12 ± 10.53% of the control) (Fig. 5E and F). On the contrary, the activity of CS, IDH, PDH, COX I, and COX II in primary hepatoma carcinoma cells was increased compared to the control group (1.72 ± 0.11, 1.16 ± 0.04, 1.35 ± 0.16, 1.26 ± 0.06, and 1.42 ± 0.13‐fold of the control) (Fig. 5F–H). These results further confirm the anticancer effect of OA in primary hepatoma carcinoma cells.

### Effects of OA on the Akt/HIF signaling pathway

**Figure 6:**
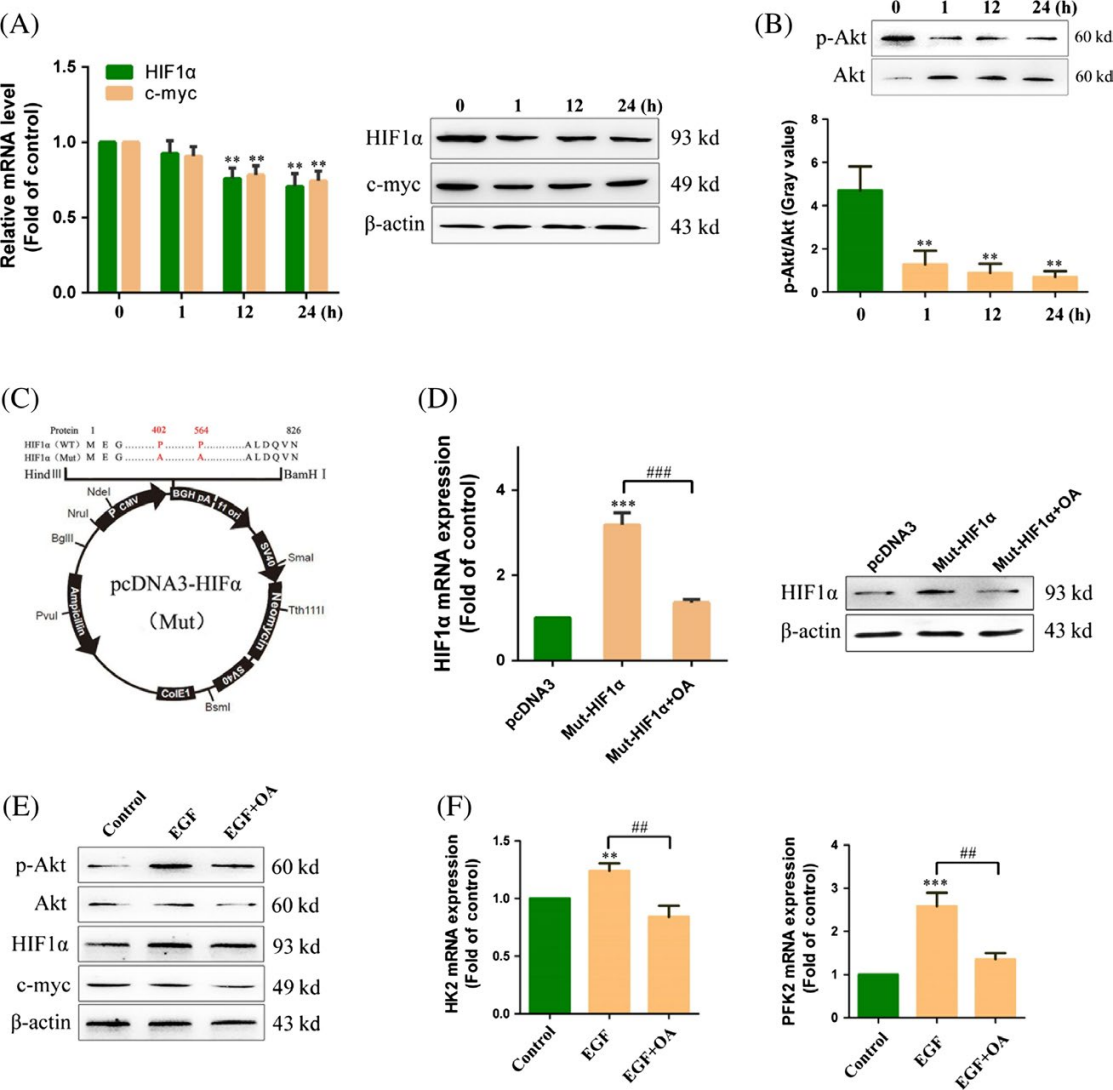
Oxaloacetate (OA) inhibited the Akt/HIF pathway in HepG2 cells. (A) HepG2 cells were treated 50 mmol/L OA for 1, 12, and 24 h. The mRNA expression and protein expression of HIF1α and c‐myc were detected using qRT‐PCR and Western blot. (B) The protein expression of Akt and p‐Akt was detected using Western blot. Gray value was detected using Image J software. (C) HIF mutant plasmid profiles. The 564th alanine and 402nd alanine were both mutated to proline. (D) HepG2 cells were transfected the HIF1α mutant plasmid and then treated with 50 mmol/L OA for 24 h. The mRNA expression and protein expression of HIF1α were detected using qRT‐PCR and Western blot. (E) HepG2 cells were stimulated by 20 ng/mL EGF for 24 h and then treated 50 mmol/L OA for 24 h. The protein expression of p‐Akt, Akt, HIF1α, c‐myc, and α‐actin was detected using Western blot. (F) The mRNA expression of HK2 and PFK2 was detected using qRT‐PCR. **P < 0.01, ***P < 0.001 versus control. ^##^ *P* < 0.01 versus EGF group. ^###^ *P* < 0.001 versus Mut‐HIF1α group. The results expressed as the mean ± SD of three independent experiments.

To investigate the molecular mechanism by which OA inhibited glycolysis in HepG2 cells, we investigated the Akt/HIF signaling pathway. The results showed that the mRNA expression and protein expression of HIF1α and c‐myc were decreased compared to the control (0 h) in HepG2 cells treated with 50 mmol/L OA for 12 and 24 h (Fig. 6A). Meanwhile, OA caused a reduction in Akt phosphorylation in HepG2 cells (Fig. 6B). To further confirm the suppression of OA on HIF1α, we transfected a HIF1α mutant plasmid into HepG2 cells (Fig. 6C). The mutant HIF1α protein was able to stably exist under normoxic conditions. The results showed that the mRNA expression and protein expression of HIF1α were increased in HepG2 cells transfected with the HIF1α mutant plasmid. We then added 50 mmol/L OA to HepG2 cells transfected with the HIF1α mutant plasmid. The results showed that the mRNA expression and protein expression of HIF1α were decreased in HepG2 cells treated with 50 mmol/L OA for 24 h (Fig. 6D). Epidermal growth factor (EGF) is an agonist of the Akt/HIF pathway. As shown in Figure 4E, the Akt/HIF pathway was activated compared to the control (0 h) in HepG2 cells treated with 20 ng/mL EGF for 24 h. However, when HepG2 cells treated with 20 ng/mL EGF plus 50 mmol/L OA, the Akt/HIF pathway was suppressed compared to the EGF‐treated group (Fig. 6E). We next detected the mRNA expression of HK2 and PFK2 in HepG2 cells treated with 20 ng/mL EGF or 20 ng/mL EGF plus 50 mmol/L OA. The results showed that EGF could up regulate the mRNA expression of HK2 and PFK2 (1.24 ± 0.06 and 2.58 ± 0.31‐fold of the control) (Fig. 6F). Compared to the EGF group, the mRNA expression of HK2 and PFK2 was decreased in HepG2 cells treated with 20 ng/mL EGF plus 50 mmol/L OA (84.13 ± 6.55 and 52.65 ± 3.18% of the EGF group) (Fig. 6F). In summary, these results indicate that OA inhibits the Akt/HIF signaling pathway, thereby inhibiting the expression of key enzymes in glycolysis.

### Selectivity of OA for the anticancer effect

**Figure 7:**
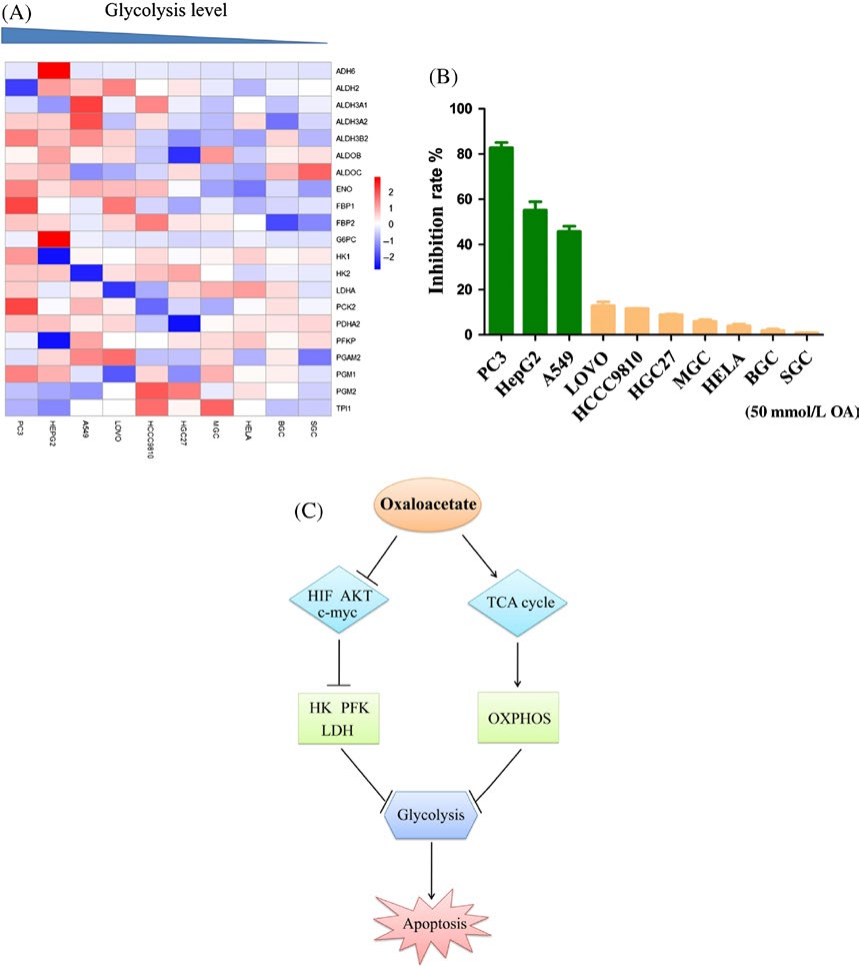
Oxaloacetate (OA) selectively inhibited highly glycolytic cancer cells. (A) Unsupervised hierarchical clustering analysis of different cancer cell lines with glycolysis‐related genes. Red keys represent values higher than the mean value of each row, and blue keys represent values lower than the mean value of each row. (B) Cancer cells with different glycolysis levels were treated with 50 mmol/L OA for 24 h. The inhibition rate of cells was measured using an MTT assay. The results expressed as the mean ± SD of three independent experiments. (C) Schematic model for the anticancer effect of OA.

In a previous study, we used HeLa cells as a cell model. We found that the anticancer effect of OA on HeLa cells was not obvious 32. Previous studies have reported that that the main energy supply in Hela cells is from the OXPHOS 13, 33. Thus, we hypothesize that the anticancer effect of OA may be related to the level of glycolysis in cancer cells. We performed unsupervised hierarchical clustering analysis with glycolysis‐related genes in different cancer cell lines. According to the expression levels of glycolysis‐related genes, we rearranged the 10 cell lines from high to low (Fig. 7A). When treated with 50 mmol/L OA for 24 h, the inhibition rate in highly glycolytic cells was obviously higher than that of lowly glycolytic cells (Fig. 7B). Even, OA had no anticancer effect on the cell lines which had low glycolysis levels. Thus, we preliminarily determine that OA selectively inhibits cancer cells that have high glycolysis levels.

## Discussion

In general, normal cells produce the majority of ATP from glucose through OXPHOS. Compared to normal cells, cancer cells have a high glucose consumption rate and produce more lactic acid despite having sufficient oxygen available to perform OXPHOS. This phenomenon is termed the Warburg effect 34. Warburg believes that cancer cells rely on glycolysis because of irreversible damage to mitochondria 35, 36. However, the view of Warburg on cancer cell mitochondria has always been controversial. Many cancer cells with high proliferative rates have been confirmed that their OXPHOS function is intact. Some studies report that if glycolysis in cancer cells is suppressed, the mitochondrial OXPHOS can be restored 37, 38. Here, we present a hypothesis that the glycolysis of cancer cells is inhibited by restoring or improving OXPHOS in cancer cells. It is reported that rapid growth of cancer cells depends on rapid energy supply from the glycolysis that can improve the tolerance of cancer cells in hypoxia through preventing OXPHOS‐induced apoptosis 39. In addition, metabolites and enzymes in the glycolysis control proliferation and apoptosis of cancer cells. For example, lactic acid produced from the glycolysis acidifies microenvironment of cancer cells to facilitate the proliferation, invasion, and migration of the cancer cells 40, and HK combines to a voltage‐dependent anion channel (VDAC) in the mitochondrial outer membrane to inhibit the apoptosis of the cancer cells 41. Therefore, inhibiting the glycolysis of cancer cells is a novel strategy to develop anticancer drugs such as 2‐deoxyglucose, lonidamine, 3‐bromopyruvate 42. In our study, we found that OA induced apoptosis in HepG2 cells in vivo and in vitro. Meanwhile, we found that OA could reduce energy metabolism in cancer cells. We further found that addition of OA could increase the level of OXPHOS and inhibit glycolysis to some extent in HepG2 cells. We believed that OXPHOS was able to reverse control glycolysis in cancer cells. The inhibition of aerobic glycolysis caused an insufficient supply of ATP in cancer cells, and ATP could not meet the rapid growth of cancer cells and cause apoptosis.

It is well understood that oxaloacetic acid is transported into the mitochondrial matrix via the malate shuttle system in the form of malate or aspartate 43, 44, 45. It is interesting that both malate and aspartate have no anticancer effect on cancer cells. We hypothesize that OA may act as a signaling molecule to regulate signaling pathways. In our study, we found that the mRNA expression and protein expression of HIF1α and c‐myc were significantly decreased in HepG2 cells treated with 50 mmol/L OA. Meanwhile, Akt phosphorylation levels were decreased in HepG2 cells treated with 50 mmol/L OA. We further confirmed the inhibitory effect of OA on the Akt/HIF pathway through transfection with a HIF1α mutant plasmid and EGF stimulation. Inhibition of Akt/HIF pathway led to a decreased expression of glycolytic‐related enzymes. Thus, we draw a conclusion that OA inhibited the glycolysis via suppression of the Akt/HIF pathway. The findings in our study suggest that OA plays a role in the signal transduction of the cell in addition to the regulation of glucose metabolism as a metabolic substrate. It is reported that 2 mmol/L OA can promote mitochondrial biogenesis 24 and 30 mmol/L OA can promote growth of hepatocytes 46. In the two reports, OA acting as a signaling molecule is suggested. Concerning alternative roles of OA, we found two other similar reports. One shows that lower concentrations of OA have an inhibitory effect on three HIF prolyl 4‐hydroxylases (P4Hs) (IC_50 _= 1, 3.8, 1.2 mmol/L) and HIF asparaginyl hydroxylase (FIH) (IC_50 _= 1.4 mmol/L) which would stabilize HIF and increase activity 47. Another one shows that 1 mmol/L OA binds to the 2‐oxoglutarate site of the HIF‐1α prolyl hydroxylases and induces inactivation of HIF‐1 hydroxylation 48. The results in the present study seem to be contradicted to those in these reports. This may reflect two faces of OA at lower or higher level in the cell in terms of the regulation of signal transduction or HIF proteins.

In our study, we used the HepG2 cell line as a cell model. OA showed an efficient anticancer effect in HepG2 cells. However, our previous research indicated that OA had no obvious inhibitory effect in HeLa cells 32. Therefore, we suggest that the anticancer effect of OA has certain selectivity. We selected ten cancer cell lines with different glycolysis levels through bioinformatics. We found that the inhibitory effect of OA in various cell lines was significantly different. We further found that highly glycolytic cancer cells were more sensitive to OA. Thus, we preliminarily concluded that OA selectively inhibited cancer cell lines with high glycolysis. Given the complex metabolic patterns of cancer cells, we would clarify the selectivity of OA in future experiments. Our previous study reported a cytotoxic effect of oxaloacetate on HepG2 cells via apoptosis and ROS generation 32. The present study validates and extends the previous study. We demonstrated that 50 mmol/L OA induced apoptosis in HepG2 cells through a mechanism of inhibition of the glycolysis by enhancement of the OXPHOS as well as suppression of the Akt/HIF pathway.

Recent studies have confirmed that OA can enhance bioenergy flow and upregulate some biosynthesis in the brain 24. Thus, OA may be applied to the treatment of Alzheimer's disease (AD). A recent preclinical study has shown that 100 mg OA capsules twice per day for 1 month is safe in patients with AD 49. In addition to the application of OA on AD, recent preclinical researches have also identified the role of OA in the treatment of diabetes, traumatic brain injury, stroke, glioma, and amyotrophic lateral sclerosis 50, 51, 52. However, there is no report on the anticancer effect of OA. Here, we found that OA could induce cancer cells apoptosis via inhibition of glycolysis in vivo and in vitro, which is a novel finding and a contribution to the treatment of cancers.

## Consent for Publication

Written informed consent was obtained from all patients.

## Ethics Approval and Consent to Participate

This study was approved by the Ethics Committee of the School of Basic Medical Sciences, Jilin University, and prior informed consent was obtained from all patients.

## Conflict of Interest

The authors declare no competing interests.
